# On Hemangioblasts in Chicken

**DOI:** 10.1371/journal.pone.0001228

**Published:** 2007-11-28

**Authors:** Wei Weng, Erike W. Sukowati, Guojun Sheng

**Affiliations:** Laboratory for Early Embryogenesis, RIKEN Center for Developmental Biology, Kobe, Hyogo, Japan; Ordway Research Institute, United States of America

## Abstract

Hemangioblasts are bi-potential precursors for blood and endothelial cells (BCs and ECs). Existence of the hemangioblast in vivo by its strict definition, i.e. a clonal precursor giving rise to these two cell types after division, is still debated. Using a combination of mitotic figure analysis, cell labeling and long-term cell tracing, we show that, in chicken, cell division does not play a major role during the entire ventral mesoderm differentiation process after gastrulation. One eighth of cells do undergo at least one round of division, but mainly give rise to daughter cells contributing to the same lineage. Approximately 7% of the dividing cells that contribute to either the BC or EC lineage meet the criteria of true hemangioblasts, with one daughter cell becoming a BC and the other an EC. Our data suggest that hemangioblast-type generation of BC/EC occurs, but is not used as a major mechanism during early chicken development. It remains unclear, however, whether hemangioblast-like progenitor cells play a more prominent role in later development.

## Introduction

During pre-circulation development in chicken, clusters of blood island cells give rise to both the vascular plexus and primitive blood cells [1], [2]. Because not all blood islands generate BCs, Sabin used the term “angioblast” to describe the blood island [3]–[5], which was later changed into “hemangioblast” by Murray [6]. Murray's hemangioblast, however, was meant to describe a cell population with the potential to differentiate into both BCs and ECs. This definition was used in most of the later descriptions of early hematopoiesis and vasculogenesis in chicken and other vertebrates [7]–[9] (and references therein). More recently, work on in vitro differentiation of stem/progenitor cells has indicated the existence of clonal hemangioblasts [10]–[15], with in vivo lineage tracing analyses in zebrafish embryos supporting the presence and importance of such hemangioblasts [16], and in mouse arguing against its importance in early development [17].

## Results

**Figure 1:**
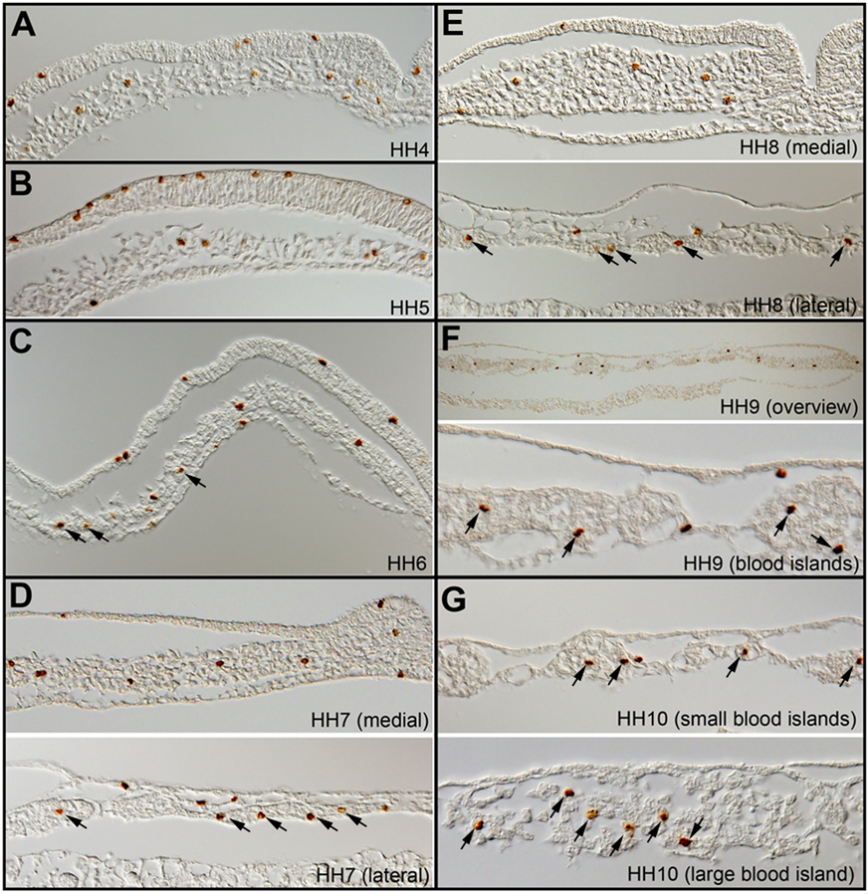
Mitotic profile from stage HH4 to HH10 by phospho-S10-H3 staining. A) HH4; B) HH5; C) HH6. Arrows indicate mitotic cells in forming blood islands; D) HH7; E) HH8; F) HH9; G) HH10. Arrows in D–G indicate dividing blood island cells.

**Figure 2:**
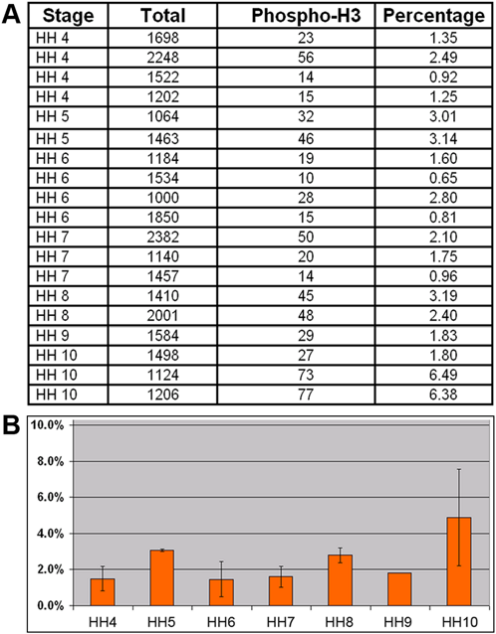
Percentage of mitotic cells in ventral mesoderm population. A) Numbers of mitotic cells scored in 19 embryos from HH4-10. For HH4-5 embryos, cells in the lateral half of the mesoderm population from the posterior half the embryo are scored. For HH6-10 embryos, mesoderm cells in blood-island forming lateral region are scored. B) Statistical representation of mitotic index in ventral mesoderm population from HH4-10.

We decided to investigate the issue of hemangioblast by its strict definition during early hematopoietic and vascular development in chicken. In this work, we use “blood island” or “blood island cells” to describe Murray's hemangioblast, and use “hemangioblast” or hemangioblasts” to describe the strict clonal definition being debated in recent literature. Since cell division is a necessary step for hemangioblast-type generation of BCs and ECs, we first analyzed the extent of mitosis taking place after the ingression of ventral mesoderm cells at stage HH4 until stage HH10 when morphologically distinct BCs and ECs can be recognized. We stained embryos with an antibody against phosphorylated Histone H3 (anti-phospho-S10-H3), which marks mitotic cells from late G2-phase to early telophase [18]. Mitotic cells were seen in all three germ layers (Fig. 1). Within the ventral mesoderm population, from which BCs and ECs are generated, cell divisions are not restricted to any particular region or stage (Fig. 1). Mitoses were seen in undifferentiated ventral mesoderm cells at HH4-6 (Fig. 1A–C), in blood-island cells prior to terminal differentiation (Fig. 1C–E; arrows) and in BC or EC populations at HH 9–10 after terminal differentiation (Fig. 1F,G; arrows). Statistical analyses revealed that approximately 2% ventral mesoderm cells were positive for phospho-S10-H3 staining at all stages (Fig. 2A,B).

**Figure 3:**
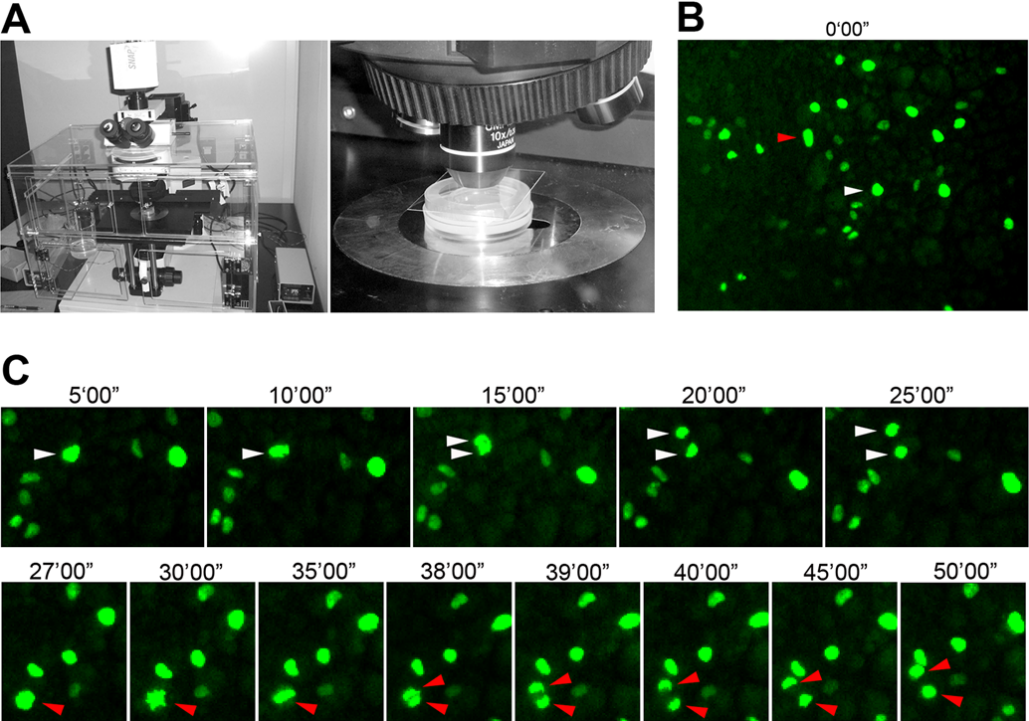
Time-lapse imaging of cell divisions in the ventral mesoderm population. A) Imaging set-up. B) Overview of a field of ventral mesoderm cells with a few dozen labeled cells. C) Two cells marked in B (red and white arrowheads) undergo division. Actual film was taken with one minute intervals.

**Figure 4:**
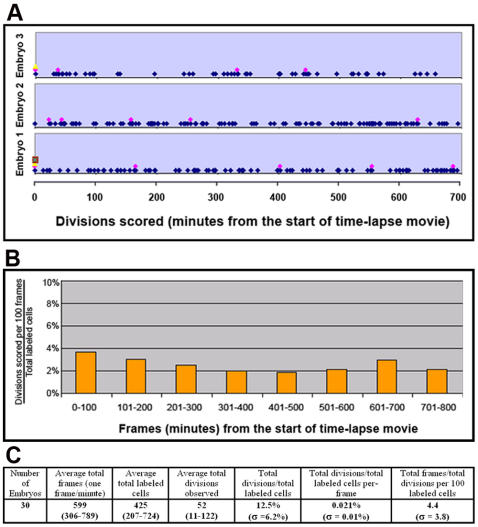
Summary of mitoses observed during time-lapse imaging. A) Three examples of scored mitoses throughout 11-hour live imaging. Images were captured with the frequency of one frame per minute. Multiple dots in a given frame represent multiple mitoses. B) Division rate (as a percentage of total labeled cells) in any given 100 frames (100 minutes). C) Summary.

The percentage of mitotic cells (so-called mitotic index), however, has to be viewed in connection with the knowledge of cell cycle duration and period of cell cycle marked by phospho-S10-H3, neither of which has been studied for the ventral mesoderm population. Similar analyses on the ectoderm, streak and general mesoderm populations during gastrulation gave a broad range of 2–10 hours for cell cycle duration and 2–8 hours for S phase duration [19]–[30]. In order to know what percentage of total ventral mesoderm cells undergo at least one round of cell division prior the separation of primitive BCs and ECs at HH9-10, we introduced an expression construct for Histone H2-GFP fusion protein into ventral mesoderm at stage HH3-4, and followed labeled cells (nuclei) by time-lapse imaging. The number of labeled cells was adjusted by varying the concentration of DNA and the size of electrode to yield ideal density for imaging. A few hours after electroporation, when labeled ventral mesoderm cells started migration toward the extraembryonic region, divisions can be readily observed and traced using time-lapse microscopy (Fig. 3A–C). Because the electroporation was targeted to the posterior primitive streak, divisions observed in the extraembryonic region represented only those in the mesoderm population, not in the ectoderm or endoderm cells. Typical anaphase lasted for about 10 minutes (Fig. 3C), and for quantification purpose we scored the mitosis (cell division) when chromatids separate at the beginning of anaphase. Each embryo had on average 400 labeled cells, and each time-lapse movie lasted 600 frames (one frame per minute) (Fig. 4C). Divisions were seen to spread out evenly throughout the tracing period (Fig. 4A,B), in agreement with the phospho-S10-H3 staining pattern. A total of 30 embryos were analyzed with 1.3×10^4^ total labeled cells and 1.8×10^4^ total frames. Approximately 12.5% of GFP-labeled cells underwent mitosis within the time-lapse period, which was typically from HH5-6 to HH9-10 (Fig. 4C). This means that about 0.02% of cells can be seen undergoing mitosis in any given minute. Taken together with phospho-S10-H3 labeling results, this suggests that phospho-S10-H3 marks about 100 minutes of the total cell cycle duration.

**Figure 5:**
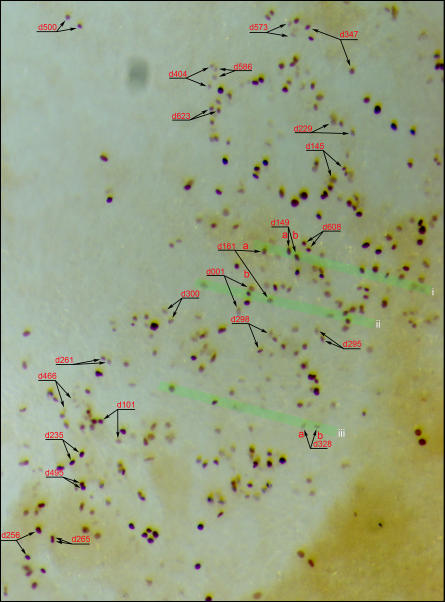
Tracking of labeled cells. Labeled cells (dividing and post-division) were tracked throughout the time-lapse imaging process and embryos were processed for anti-GFP staining immediately after the last frame. Successfully tracked and matched daughter pairs are shown in this example (whole-mount, anti-GFP stained embryo). The area shown is located in the right-lateral and posterior region of an HH10 embryo. Each daughter pair is also marked with the time of observed mitosis (e.g., d161 represents the division observed at the 161th minute of filming). Three green highlighted stripes (i, ii and iii) indicate regions of sections shown in Fig. 6A and B.

**Figure 6:**
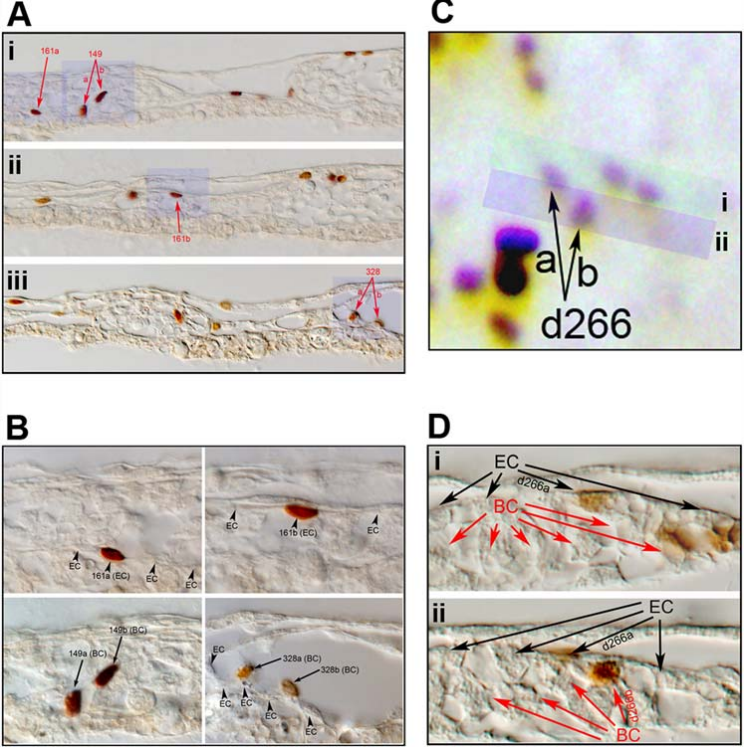
Most divisions have daughter cells of the same fate. A) Section view of three regions indicated in Figure 5 (i, ii and iii). Three divisions (d149, d161 and d328) give rise to 2 BC pairs (d149 shown in Fig. 6Ai; d328 shown in Fig. 6Aiii) and one EC pair (d161 shown in Fig. 6Ai and 6Aii). B) Magnified view of blue highlighted region in A. Arrowheads: ECs; Arrows: BCs. C) Occasional hemangioblast-type divisions are seen, represented by d266 (with d266a becoming EC and d266b becoming BC). D) Section view of highlighted regions in C (i and ii). Black arrows: ECs; Red arrows: BCs.

In order to assess the fate of daughter cells from each division, we tested the possibility of tracking the daughter cells of all divisions, regardless of when they take place, to the end of entire time-lapse period. Frame-by-frame analyses suggested that this was difficult to achieve and we lost track of most daughter pairs at some point for two main technical reasons: 1) short periods of being slightly out of focus; 2) migration of these cells close to other labeled cells. Nonetheless, out of approximately fifteen hundred total divisions observed, we were able to track 221 daughter pairs with 100% confidence to the end of time-lapse period. To assess whether any of them could be progenies of the hemangioblast, we fixed the embryos immediately after time-lapse tracking and processed them for anti-GFP antibody staining, followed by paraffin sectioning of each embryo. Based on the final frames of time-lapse tracking, daughter pairs in whole-mount anti-GFP stained embryos were marked as shown in Figure 5. GFP-labeled cells in sections were then carefully matched to those in the whole-mount. Of 221 daughter pairs, we were able to match 105 with 100% confidence in final sections. Among them, 24 pairs became ECs (Fig. 6A,B), 28 BCs (Fig. 6A,B) and 4 of the hemangioblast type (Fig. 6C,D). The remaining 49 pairs were of undetermined contribution, mainly due to insufficient resolution to assign a BC or EC fate, and to the contribution to non-BC/EC cell types in the extraembryonic mesoderm. These numbers suggest that, among dividing ventral mesoderm cells contributing the BC or EC fate, about 7% are hemangioblasts.

## Discussion

Our analyses indicate that hemangioblasts exist in the early chicken embryo, but that they do not serve as a main mechanism to generate BCs and ECs during primitive hematopoiesis and vasculogenesis. We observed that about one eighth of labeled ventral mesoderm cells undergo division before reaching stage HH10. Even if we factor in the variable length of tracking time (average 10 hours with a maximum of 13 hours, in comparison with 15–20 hours of normal development time from HH4-HH10), at least three quarters of ventral mesoderm cells do not divide throughout the entire differentiation process, suggesting that the original definition of hemangioblast (as a population of cells with dual potentials) by Murray may be more appropriate in the context of early development in chicken. In addition, on two occasions when we observed a second round of division of one of the daughter pair, we were able to calculate the cell cycle time to be 510 and 600 minutes. Taken together, our data may be interpreted to reconcile contradictory results from analyses in mouse and zebrafish embryos. On the one hand, the rarity of hemangioblasts we have seen is in agreement with the mouse analysis [17]. On the other hand, among cells that do divide, we show that 7% of BC or EC contributing cells are of the hemangioblast type, a number comparable to what was obtained from the zebrafish analysis (12.5%) [16]. The significance of hemangioblasts may be more relevant from the perspective of progenitor/stem cells. During normal ventral mesoderm development, most cells undergo terminal differentiation, with a very small percentage of them being maintained as undifferentiated mesenchymal cells (unpublished data). Among these progenitor/stem cell populations may exist potential hemangioblasts set aside for later development, similar to what has been suggested in dorsal aorta [31], the study of which will require the identification of proper molecular markers.

## Materials and methods

### Molecular reagents

Anti- phospho-S10-H3 antibody was purchased from Upstate Cell Signaling (NY, #06-570), anti-GFP antibody from Invitrogen (OR, #A11122) and secondary goat anti-rabbit HRP from DakoCytomation (Glostrup, Denmark; #0448). Histone H2-GFP fusion protein expression construct was a kind gift from Dr. Hadjantonakis (MSKCC, NY).

### Embryology and embryo processing

Fertilized chicken eggs were purchased from Shiroyama Farm (Kanagawa, Japan). Embryos were cultured in ovo to HH3-4 for GFP electroporation (Intracel electroporator; Intracel, UK), or to desired stages for phospho-S10-H3 staining. For imaging, GFP-electroporated embryos were grown in New culture setting [32] to HH5-6 and were then flipped dorsal-side up and cultured in agar-albumin setting [33] with the agar concentration lowered to 0.3%. Normal cover-glass (0.12–0.17mm thick) and sealing film (Parafilm) were used to keep the moisture inside agar-albumin culture dish during imaging. A home-made chamber, additionally moisturized with a beaker of tap water and temperature-controlled with two Thermostats (Kokensha Engineering, Tokyo, Japan), was used for incubation during live imaging. Post-imaging fixation, staining and paraffin sectioning followed normal protocols [34]. Care was taken to ensure the integrity of all sections for proper matching to the whole-mount images. Section images were taken with an Olympus BX51 microscope fitted with DP70 camera (Olympus, Japan).

### Imaging

Time-lapse imaging and tracking were carried out with an Olympus BX51 microscope (Olympus, Japan), fitted with long working distance 10X objective (NA = 0.3 and WD = 6.5 mm) and occasional use of 50X objective (NA = 0.5 and WD = 10.6 mm), cool CCD camera and Metamorph software (Olympus, Japan). Images were taken with one minute intervals and 100–500 ms exposure, and the focus was manually adjusted periodically.
